# Do Plasmodesmata Play a Prominent Role in Regulation of Auxin-Dependent Genes at Early Stages of Embryogenesis?

**DOI:** 10.3390/cells10040733

**Published:** 2021-03-26

**Authors:** Konrad Winnicki, Justyna Teresa Polit, Aneta Żabka, Janusz Maszewski

**Affiliations:** Department of Cytophysiology, Faculty of Biology and Environmental Protection, University of Lodz, ul. Pomorska 141/143, 90-236 Łódź, Poland; justyna.polit@biol.uni.lodz.pl (J.T.P.); aneta.zabka@biol.uni.lodz.pl (A.Ż.); janusz.maszewski@biol.uni.lodz.pl (J.M.)

**Keywords:** auxin, plasmodesmata, calcium, embryogenesis, cellular patterning, callose, cell-to-cell communication, ARF, AUX/IAA, ABP1

## Abstract

Plasmodesmata form intercellular channels which ensure the transport of various molecules during embryogenesis and postembryonic growth. However, high permeability of plasmodesmata may interfere with the establishment of auxin maxima, which are required for cellular patterning and the development of distinct tissues. Therefore, diffusion through plasmodesmata is not always desirable and the symplastic continuum must be broken up to induce or accomplish some developmental processes. Many data show the role of auxin maxima in the regulation of auxin-responsive genes and the establishment of various cellular patterns. However, still little is known whether and how these maxima are formed in the embryo proper before 16-cell stage, that is, when there is still a nonpolar distribution of auxin efflux carriers. In this work, we focused on auxin-dependent regulation of plasmodesmata function, which may provide rapid and transient changes of their permeability, and thus take part in the regulation of gene expression.

## 1. Introduction

Expression of auxin-responsive genes seems to rely on a multilevel regulatory machinery, and it depends not only on sophisticated molecular mechanisms controlling transcription, but also on systems which underlie the establishment of auxin maxima. Auxin controls the expression of various genes in a threshold-dependent manner, and some of them were found to be upregulated while others repressed in response to a high auxin concentration [1,2,3]. Auxin synthesis starts in the apical region of the 16-cell embryos; however, a strong response to auxin was found in hypophysis. Auxin maxima are formed due to polar auxin transport (PAT), which relies on auxin influx and efflux transporters localized in the plasma membrane. The former group includes AUXIN RESISTANT1/LIKE AUX1 (AUX1/LAX) transporters, the latter consists of ATP-BINDING CASSETTE subfamily B (ABCB) proteins and PIN-FORMED (PIN) carriers [4,5,6,7,8]. PIN polarization results from phosphorylation and dephosphorylation driven by PINOID protein kinase and PP2A phosphatase, respectively. Phosphorylated PIN proteins occupy the apical domain of the cell, and their dephosphorylation results in trafficking to the basal plasma membrane. However, PIN phosphorylation performed by D6 PROTEIN KINASE (D6PK) regulates auxin efflux activity without affecting protein location [9,10,11]. Mutations in genes responsible for PAT disrupt the formation of the apical-basal axis and may be lethal for the developing embryo [8,12,13]. Thus, the establishment of auxin maxima seems to be crucial during changes of the radial to bilateral symmetry.

On the contrary, before embryos reach the 16-cell stage, similar auxin concentrations are considered in all cells of the embryo proper due to nonpolar distribution of auxin efflux carriers [14,15]. Expression of the auxin-responsive *DORNRÖSCHEN* (*DRN*) gene [6,16] and activation of the DR5 promoter [8] in each cell of the embryo proper may support a homogenous auxin dispersion. However, cell-specific activation of auxin-dependent genes was found early during embryogenesis. After the first division of a zygote different genes are expressed in the apical cell rather than in the basal one, and this pattern is maintained during the following stages of embryo development. Auxin carriers and transcription factors belonging to WUSCHEL-related homeobox (WOX), homeodomain-leucine zipper class Ⅲ (HD-ZIP Ⅲ), or Apetala2 (AP2)-domain families are good examples of proteins being expressed differently in various regions of the developing embryo [12,17,18,19,20]. Thus, auxin threshold-dependent regulation of cell identity may occur after the first division of a zygote. However, one may ask whether various intracellular auxin concentrations may be established in an embryo proper before it reaches the 16-cell stage, that is, when there is still a nonpolar distribution of auxin efflux transporters.

A hypothesis can be put forward that the increase in the auxin level and the differences in their concentrations between the upper and lower tier of the 8-cell embryos may be transient, but long enough to initiate a distinct fate of these two embryonic regions. Based on the available data concerning the control of auxin-dependent genes during embryogenesis and post-embryonic growth, in this review we sketched possible molecular pathways which could regulate the expression of auxin-dependent genes in embryos before they reach the 16-cell stage. Furthermore, different regulatory mechanisms of plasmodesmata permeability were overviewed, pointing to these which possibly induce transient differences in auxin concentrations between the upper and lower tier during early stages of embryogenesis.

## 2. Multi-Level Regulation of HD-ZIP Ⅲ and AP2-Domain Transcription Factors

HD-ZIP Ⅲ and AP2-domain transcription factors are expressed in distinct cell populations during proembryo development. *PLETHORA1 (PLT1)* transcripts, coding one of the AP2-domain transcription factors, were found in the lower tier already at the 8-cell stage [21,22]. At the same time, antagonistically acting PHABULOSA (PHB), belonging to HD-ZIP Ⅲ transcription factors, appears in the upper tier [23]. WOX2, which was found in an apical cell after zygote division, seems to regulate *PHB* synthesis in this region. [24]. Lack of PLT transcription factors in the upper tier may result from AUXIN RESPONSE FACTOR (ARF) repression driven by non-canonical AUXIN/INDOLE-3-ACETIC ACID proteins (AUX/IAAs). This finding may be further supported by the fact that seedlings of *Arabidopsis thaliana* overexpressing IAA20 and IAA30 resemble *PLT* mutants [25]. Furthermore, the expression of these ARF repressors seems to be controlled by PHB protein [26]. Interactions between non-canonical AUX/IAAs and ARFs may also be regulated at the level of posttranscriptional modifications, and phosphorylation driven by TRANSMEMBRANE KINASE1 (TMK1) and MITOGEN-ACTIVATED PROTEIN KINASE14 (MPK14) was found to modulate the repressor function of non-canonical AUX/IAAs. Interestingly, AUXIN BINDING PROTEIN1 (ABP1), an apoplast-localized auxin receptor, may induce activation of the TMK1/MPK14-signaling pathway [27,28]. Although IAA20 and IAA30 were not revealed in the embryo proper at the 8-cell stage [29], Llavata–Peris and coworkers suggested that IAA33, another non-canonical AUX/IAA, was expressed in all cells until the late globular stage [30]. Thus, it is possible that the regulation of the expression of non-canonical AUX/IAAs and their phosphorylation take part in the repression of *PLT* genes in the upper tier at early stages of embryogenesis.

**Figure 1:**
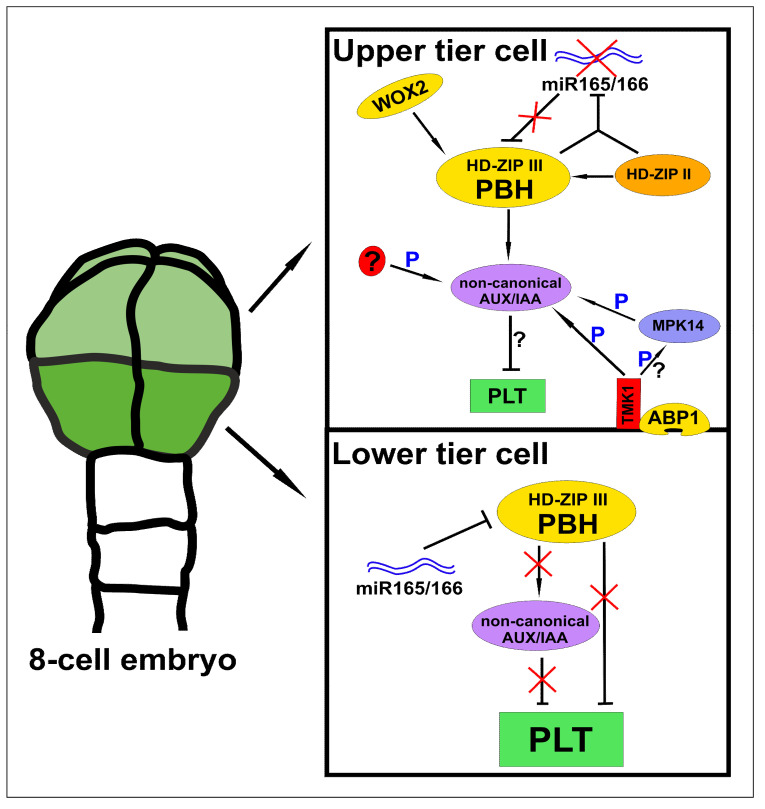
Molecular pathways which may underlie the differential expression of homeodomain-leucine zipper class Ⅲ (HD-ZIP Ⅲ) and Apetala2 (AP2)-domain family transcription factors in the upper and lower tier of 8-cell embryos. In the upper tier, PHABULOSA (PHB), which belongs to HD-ZIP Ⅲ family transcription factors, may regulate the expression of non-canonical AUXIN/INDOLE-3-ACETIC ACID proteins (AUX/IAA) and thus support their high concentration. Phosphorylation of non-canonical AUX/IAAs (indicated as P), which underlies their stability, may be performed by TRANSMEMBRANE KINASE1 (TMK1), MITOGEN-ACTIVATED PROTEIN KINASE14 (MPK14), or other kinases. High level of phosphorylated AUX/IAAs blocks the expression of AP2-domain transcription factors. In the lower tier, miRNAs downregulate the expression of HD-ZIP Ⅲ transcription factors, and therefore do not support enhanced expression of non-canonical AUX/IAAs at this stage of embryogenesis. Absence of HD-ZIP Ⅲ proteins and the possible low concentration/phosphorylation of specific non-canonical AUX/IAAs may allow for the expression of AP2-domain transcription factors in the lower tier.

The activity of microRNAs (miRNAs) may be an additional yellow brick road which leads to better understanding how the expression of HD-ZIP Ⅲ and AP-2 domain transcription factors is established in various regions of developing embryos. Synthesis of HD-ZIP Ⅲ family proteins may be affected by miR165 and miR166, both expressed exclusively in suspensor cells at the 8-cell stage. Thus, lack of *PHB* transcript synthesis in the lower tier could result initially from transport of miRNAs to the embryo proper. Nevertheless, at the 16-cell stage, miR166 is also expressed in cells of the lower tier [23]. Significance of miRNAs to correct spatio-temporal expression of antagonistically acting HD-ZIP Ⅲ and AP-2 domain transcription factors was indicated in other studies as well. Embryos with the mutation in the *ARABIDOPSIS SUPPRESSOR OF TY INSERTION 6-LIKE* (*SPT6L*) gene, which encodes proteins necessary for miRNA-mediated gene silencing, show lack of *PLT1* expression and ectopic synthesis of *PHB* transcripts in the basal region of an embryo. Interestingly, *SPT6L/PHB/PHV* mutants display partially restored expression of the *PLT1* gene [31]. Thus, it is possible that lack of PLT proteins in the upper tier results either from blockage driven directly by HD-ZIP Ⅲ proteins or indirectly through non-canonical AUX/IAA repressors or other not yet revealed factors. Synergistic action of various pathways cannot also be excluded (Figure 1).

All of this indicates that the mechanisms underlying the expression of HD-ZIP Ⅲ and AP2-domain family proteins at the 8-cell stage still need elucidation. Since these antagonistically acting protein families are encoded by auxin-dependent genes, different auxin concentrations may underlie various expression patterns. Although PAT, supported by auxin efflux carriers, is not yet established at the 8-cell stage, different intracellular auxin concentrations could result from changes in the plasmodesmata (PD) permeability. The paragraphs below provide an overview of the functional connection between the establishment of auxin maxima and the auxin-dependent control of PD aperture.

## 3. The Role of Plasmodesmata during Developmental Processes

Many developmental processes in plants rely on cell-to-cell communication, which is ensured by channels called plasmodesmata (PD). These intracellular pores show a far-reaching structural complexity, and many proteins responsible for their function have been described so far. The presence of a structural and functional symplastic continuum is crucial for the transport of signaling molecules, phytohormones, RNAs, and proteins [32,33,34,35]. Plants modify both the number of PD and the size of their aperture to regulate complicated developmental processes during ontogenesis and to provide a proper response to pathogens [36,37,38]. PD may already appear after the first division of a zygote [39]; thus, the regulation of their function plays a crucial role in the establishment of various cellular patterns during plant embryogenesis. In *A. thaliana*, the embryonic root patterning relies on TARGET OF MONOPTEROS 7 (TMO7) protein diffusion from cells of the lower tier of the embryo proper to the hypophysis, the uppermost suspensor cell. Interestingly, this protein does not appear in the upper tier of the embryo proper, indicating a directional hypophysis-oriented transport in this case [40,41]. The symplastic continuum is not always desirable for the correctness of the developmental processes and at the early heart stage the hypophysis was found to be isolated from other suspensor cells due to changes of the PD aperture. However, this isolation seems to be unidirectional, and although the proteins synthesized in the suspensor do not appear in the embryo proper, those expressed under the *SHOOTMERISTEMLESS (STM)* promoter are present in suspensor cells. A far-reaching isolation of embryo regions is established at the cotyledonary stage, and PD permeability is reduced for high molecular mass proteins in this case [42,43,44]. Although the symplastic continuum is beneficial under some circumstances, it seems that the cell-to-cell communication must be broken up not only at the heart stage, when different tissues and distinct regions of an embryo are formed, but also at early stages of embryogenesis when cellular patterning starts.

## 4. Do Callose-Dependent Changes of PD Permeability Regulate Transient Auxin Gradients?

Dysfunction of PD leads to severe developmental disorders during embryogenesis and postembryonic growth, indicating that the on-time regulation of PD permeability is crucial for various cellular processes [45,46,47]. During postembryonic growth, PD were proposed to provide auxin reflux from shootward transporting tissues, which helps in the establishment of an auxin sink in the root apical region [48]. Although it is possible that the reflux model plays a similar role during auxin accumulation in a radicle, auxin diffusion does not seem to be helpful during the establishment of various cellular patterns in proembryo cells. To counteract this and provide auxin maxima in particular cells during early stages of embryogenesis, dynamic and reversible changes of PD permeability are needed. Thus, it is reasonable to ask whether auxin may control PD permeability to support the establishment of an increased auxin concentration in various cells and tissues.

Several studies indicate that deposition of 1,3-β-glucan (callose) decreases the PD aperture during ontogenesis and in response to pathogens. The mutation in the *GLUCAN SYNTHASE-LIKE 8 (GLS8)* gene, which encodes a callose synthetase, leads to severe aberrations during embryo development. Callose-dependent changes of the PD aperture are responsible for the directional diffusion of auxin in midrib and petiole epidermis cells [49], and higher PD permeability for auxin was seen in *gsl8* mutants, compared to wild-type plants [50]. Callose synthesis was also found to be indispensable during the formation of the apical-basal axis and the development of the embryonic root [45,46,51,52].

The establishment of auxin gradients during phototropic and gravitropic responses rely on callose-dependent PD closure, and high auxin levels were found to activate the *gls8* gene. Furthermore, the expression of callose synthetase is regulated by ARF7 [50]. Auxin’s role in the regulation of PD aperture may be supported by the data which indicate that expression of PLASMODESMATA-LOCATED PROTEIN 5 (PDLP5) in cells surrounding the lateral root primordium requires derepression of ARF7/19 transcription factors. PDLP5 is a receptor-like transmembrane protein which stimulates PD closure in a callose-dependent manner [52]. All of this indicates that auxin may control the PD aperture due to callose deposition. However, does it ensure fast regulation of PD permeability which would be needed for dynamic processes during early stages of embryogenesis?

Although rapid callose deposition was found during a bacterial pathogen attack [53], returning to the initial state, which is necessary for transient changes of the PD aperture, requires the activity of glucanases, the enzymes responsible for the degradation of callose [54,55,56]. Glucanases, similarly to callose synthetase, were found to be regulated in an auxin-dependent manner [57,58]; however, callose synthesis and turnover do not seem to support dynamic changes of PD permeability. Thus, the auxin-dependent deposition of callose in PD may play a main role in slow developmental processes or plant responses which do not require fast and reversible modification of the PD aperture. It is reasonable to hypothesize that the regulation of PD permeability, which underlies the dynamic processes of cellular patterning during early stages of embryogenesis, might occur in a callose-independent manner.

## 5. Auxin May Regulate the Function of PD in a Callose-Independent Manner

**Figure 2:**
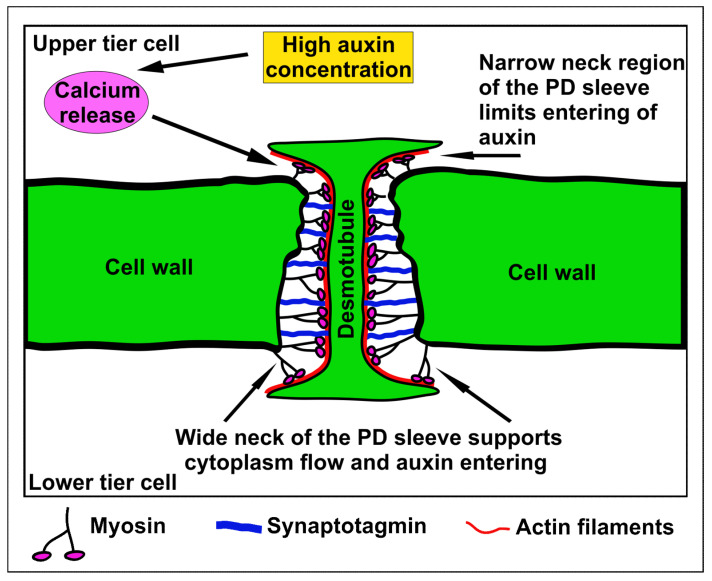
A hypothetical model of rapid plasmodesmata aperture regulation. The auxin influx may induce calcium ion release in the upper tier cells. Next, a high calcium concentration in the cytoplasm triggers changes in the conformation of myosin and synaptotagmin proteins, which reduce the aperture of the neck region at one side of the plasmodesma. This could favor unidirectional auxin movement, which may underlie the establishment of different auxin maxima in adjacent cells. This mechanism could also induce a reduction of cell-to-cell communication if calcium release happens in both cells.

In the previous century, a calcium-dependent PD closure was observed in the staminal hairs of *Setcreasea purpurea* [59] and the role of calcium in this process was also revealed by other authors [60]. Since calmodulin, calreticulin, actin, and myosin Ⅷ were found in PD, calcium ions may regulate the organization of actin filaments and myosin motility properties which leads to changes in PD aperture. Actin–myosin structures may be localized in the neck region of PD; however, myosin Ⅷ was also postulated to form spoke-like structures which connect the plasma membrane and the desmotubule in the cytoplasmic sleeve of PD [61,62]. Additionally, other proteins such as plant synaptotagmins are suggested to form membrane contact sites (MCSs). Synaptotagmin-dependent tethering of the plasma membrane and desmotubule was reviewed by Tilsner and coworkers who proposed that calcium might reduce the distance between the plasma membrane and desmotubule similarly as it happens between the plasma membrane and endoplasmic reticulum in animal cells [63]. A high calcium concentration was found to shorten the neck domain of myosin Ⅺ in plants [64], which suggests that a similar effect of calcium could also concern myosin Ⅷ. Therefore, myosin localized in the cytoplasmic sleeve may take part in the reduction of the distance between the plasma membrane and the desmotubule. Since auxin was found to induce calcium release [65], calcium-dependent regulation of PD permeability at early stages of embryogenesis seems to be a very promising hypothesis, and this callose-independent pathway has been suggested in some review papers [38,66]. If calcium release occurs in only one of the adjacent cells, this mechanism could result in a wider opening of PD on one site of the cell wall, which helps to provide directional transport of molecules (Figure 2).

The most recent data indicate that the glucose-dependent activation of the plant TARGET OF RAPAMYCIN KINASE (TOR) restricts the transport through PD in leaf epidermal cells [67]. Interestingly, auxin was found to activate the TOR kinase via RHO of plants (ROPs), which are plasma membrane GTP-binding proteins. Another signaling pathway of TOR kinase activation is possible via phosphatidic acid (PA), which may also be formed in response to auxin. This auxin-dependent activation of TOR kinase was well reviewed by Schepetilnikov and Ryabova [68]. Since no connection between TOR activation and the deposition of callose has been shown so far, the TOR-dependent signaling pathway could function as an alternative mechanism of callose-independent regulation of PD permeability in response to auxin.

Another mechanism which could explain the transient and rapid changes in plasmodesmata permeability relies on the pressure forces generated inside the cell. In 1992, Oparka and Prior indicated that the increase in turgor pressure and differences in the pressure generated between the two cells stopped the intracellular transport [69]. Through the years, the pressure-dependent control of plasmodesmata permeability has been speculated and after over 20 years, Park and coworkers described a model explaining the regulation of PD function in a mechano-sensing manner. The authors proposed that the cytoplasmic part of desmotubules may work as a plug which closes the entrance to the cytoplasmic sleeve of PD when differences in pressure appear between cells [70]. Changes in turgor pressure may be induced by osmotic substances, and potassium ions are one of the main players which may regulate this process [71]. Auxin was found to activate channels which allow for the influx of potassium ion [72,73], and auxin-induced potassium channels seem to be indispensable for embryo development [74]. Thus, auxin-regulated variations in the turgor pressure may be responsible for rapid and transient changes of plasmodesmata permeability. Auxin may regulate this pathway already before high cellular concentration of auxin is achieved. Changes in potassium flux were indicated in cells overexpressing ABP1 [75] and after using antibodies against this protein [76]. Thus, signals which regulate the turgor pressure may appear already when auxin binds to ABP1 in the apoplast.

## 6. Unidirectional Transport through PD—Science or Fiction

In the cytoplasm, small molecules such auxin move generally via diffusion; thus, the regulation of PD permeability seems to be necessary to generate transient intracellular auxin maxima when the PAT is not yet established. It could be achieved by either PD closure in a mechano-sensing manner or by directional auxin transport resulting from changes in the structure of PD channels. Christensen and coworkers [77] observed diffusion of fluorescent probes from an epidermal cell to a trichome basal cell, but not in the opposite direction. This unidirectional transport did not depend directly on actin filaments and it was blocked by treatment with sodium azide, which is a metabolic inhibitor. Unidirectional transport was also indicated in elegant studies which showed the transfer of photoactivatable green fluorescent protein (PA-GFP) from basal to apical cell in embryos of *Nicotiana tabacum*, but not in the opposite direction [78]. One-way transport through PD may be indirectly supported by other data. GFP expressed in the base of a hypocotyl was found to diffuse into the root apical meristem (RAM) and the entire hypocotyl, while GFP synthesized in RAM was unable to cross the boundary between the root and hypocotyl [43]. All of this shows that PD by unidirectional transport could support the establishment of auxin maxima even if the system based on auxin influx and efflux transporters does not allow for this. However, the exact molecular mechanism of unidirectional transport remains to be discovered.

Another pathway which could participate in the generation of auxin maxima may be based on vesicle-dependent auxin transport [79,80,81]. Although the presence of auxin secretory vesicles was questioned by some authors [82], Hille and coworkers suggested that this vesicular trafficking was possible; however, they concluded at the same time that its role during directional transport is negligible [83]. Studies which showed no directional aggregation of endosomes until embryos reach the 16-cell stage [84] cast doubt on the importance of secretory vesicle-dependent auxin transport at early stages of embryogenesis. However, it cannot be excluded that very small secretory vesicles still function as a platform of directional auxin transport during embryo development.

## 7. Conclusions

Many developmental processes during early stages of embryogenesis may depend on the rapid and transient establishment of auxin maxima; thus, cells must counteract auxin diffusion to provide sufficient auxin concentrations. Current data indicate that auxin may regulate dynamic changes of PD permeability during various cellular processes, and the control of PD function seems to take advantage of their complex structure. Mechano-sensing and myosin-based regulation of PD permeability could play a prominent role during the establishment of auxin maxima in situations when auxin efflux transporters do not show a polar distribution.

Although the basis of callose-dependent changes of the PD aperture seems to be well understood, the dynamic regulation of their permeability in a callose-independent manner still needs elucidation. Thus, detailed studies on the regulation of auxin-responsive genes in the context of cytophysiological mechanisms responsible for the establishment of auxin gradients should be continued. The existence of yet unknown regulating systems is very plausible, and they are still waiting to be discovered.
